# Social and professional recognition are key determinants of quality of life at work among night-shift healthcare workers in Paris public hospitals (AP-HP ALADDIN COVID-19 survey)

**DOI:** 10.1371/journal.pone.0265724

**Published:** 2022-04-07

**Authors:** Martin Duracinsky, Fabienne Marcellin, Lorraine Cousin, Vincent Di Beo, Véronique Mahé, Olivia Rousset-Torrente, Patrizia Carrieri, Olivier Chassany

**Affiliations:** 1 Département de Médecine Interne et d’immunologie Clinique, Hôpital Bicêtre, AP-HP, Kremlin-Bicêtre, France; 2 Patient-Reported Outcomes Unit (PROQOL), UMR 1123, Inserm, Université de Paris, Paris, France; 3 Unité de Recherche Clinique en Économie de la Santé (URC-ECO) AP-HP, Hôpital Hôtel-Dieu, Paris, France; 4 Inserm, IRD, SESSTIM, Sciences Economiques & Sociales de la Santé et Traitement de l’Information Médicale, ISSPAM, Aix Marseille Univ, Marseille, France; 5 Service Central de Santé au Travail, Hôpitaux Lariboisière-Fernand Widal, AP-HP Nord, Paris, France; Charite Universitatsmedizin Berlin, GERMANY

## Abstract

**Objective:**

Documenting the perceptions and experiences of frontline healthcare workers during a sanitary crisis is key to reinforce healthcare systems. We identify the determinants of quality of working life (QWL) among night-shift healthcare workers (NSHW) in Paris public hospitals shortly after the first-wave of the COVID-19 pandemic.

**Methods:**

The ALADDIN cross-sectional online survey (15 June to 15 September 2020) collected QWL, socio-economic, behavioral, and work-related information among 1,387 NSHW in the 39 hospitals of the Assistance Publique—Hôpitaux de Paris (AP-HP). Data were weighted (margin calibration) to be representative of the entire population of 12,000 AP-HP hospitals’ NSHW regarding sex, age, and professional category. Linear regression was used to identify correlates of QWL (WRQoL scale).

**Results:**

New night position during the COVID pandemic, difficulties in getting screened for COVID, and considering protective measures inadequate were associated with poorer QWL, after adjustment for socio-economic characteristics, professional category, perceived health, physical activity, and history of harassment at work. Under-estimation of night-shift work by day-shift colleagues, reporting night work as a source of tension with friends, or feeling more irritable since working at night also impaired QWL. By contrast, satisfaction regarding COVID information received from the employer, and feeling valued by the general population during the pandemic improved QWL.

**Conclusions:**

Insufficient access to screening, information, and protective measures impaired QWL of NSHW after the first wave of COVID-19 in Paris public hospitals. Social and professional recognition of night-shift work were the key determinants of QWL in this population.

## Introduction

The number of individuals involved in night-shift work has increased in the last twenty years in Western countries, and people working in the healthcare sector are among the most represented [1–3]. Night-shift work negatively impacts health, notably because of circadian rhythms perturbations [4–8]. Its multiple potential consequences include fatigue, sleep disturbance, an increased risk of cancer, cardiovascular diseases, metabolic syndrome, affective disorders, and impaired cognitive function [9–19]. Beyond these negative health consequences, night-shift work has also detrimental effects on quality of life [20–22], which closely interacts with perceived health. Quality of working life (QWL), a multidimensional definition of well-being in the workplace, directly influences quality of life [23]. QWL is closely linked to factors such as workload, work-life balance, meaning of work and meaning at work [24, 25], the latter two factors distinguishing how one individual perceives the meaning of what one does at work from the group to which one self-identifies in said work environment [26]. A recent literature review targeting healthcare work showed that QWL may also influence quality of care [27]. In the COVID-19 era, QWL remains poorly documented among hospital night-shift healthcare workers (NSHW), a population exposed to a higher risk of infection [28], and who contributes to the continuity of care since the beginning of the pandemic. Managing healthcare systems during sanitary crises represents human and organizational challenges with potential mental health and quality of life implications [29]. A better understanding of QWL among NSHW is therefore needed, both from a public health perspective, and to identify levers which could help strengthening healthcare systems in such contexts.

The present study aims to document QWL among NSHW in Paris public hospitals shortly after the first-wave of the COVID-19 pandemic and to identify its determinants.

## Materials and methods

### The AP-HP ALADDIN survey

The ALADDIN cross-sectional survey (15 June 2020 to 15 September 2020) was conducted among NSHW in public hospitals in Paris. It included all 39 hospitals of the Assistance Publique—Hôpitaux de Paris (AP-HP). One of the main objectives was to document NSHW’s QWL (i.e. perceived quality of life at work) and its correlates shortly after the first wave of the COVID-19 pandemic (March to May 2020), once healthcare workers were more available to participate in the survey. All individuals working in the AP-HP hospitals with a night-shift or a day/night alternation employment contract, regardless the years of experience, working full-time or part-time, could participate in the survey. Exclusion criteria were as follows: (i) working only during the day; (ii) working less than three hours a day between 9 p.m. and 6 a.m. twice a week (including on-duty or on-call staff). In order to maintain a homogeneous study population, physicians were excluded from the analyses as they constitute a subgroup with specific characteristics. Sample size was expected to reach 10% of the 12,000 NSHW working in the AP-HP hospitals (target population).

### Ethics

The AP-HP ALADDIN survey was approved by the Lyon 2 ethics committee in March 2020 (ID RCB202-A00495-34). Informed consent was obtained for all survey participants.

### Data collection

During the AP-HP ALADDIN survey, quantitative data were collected using an online questionnaire which documented participants’ sociodemographic, economic and work-related characteristics, perceived health, QWL, as well as perceptions and experience since the beginning of the COVID-19 pandemic [30, 31]. NSHW’s perceptions regarding their social and professional recognition were assessed using items related to under-estimation of night-shift work by colleagues, loved ones, and patients; perceptions of the importance of night missions and of workload during night; feeling valued by the general population as a NSHW during the pandemic. Most of these items were derived from different stigma scales [32–34]. NSHW could respond to the questionnaire online (NetSurvey®), using either their computer at work or their personal electronic devices.

### Assessment of QWL

NSHW’s QWL was assessed using the work-related quality of life (WRQoL) scale [35] which includes 24 items, each associated with five possible answers on a Likert-type scale (strongly disagree/disagree/neutral/agree/strongly agree). The WRQoL scale explores six dimensions of quality of life related to the work environment of NSHW. Each dimension is associated with a factor score, calculated from respondents’ answers to the first 23 items of the scale: general well-being (GWB, score range: 0 to 30); home-work interface (HWI, score range: 0 to 15); job and career satisfaction (JCS, score range: 0 to 30); control at work (CAW, score range: 0 to 15); working conditions (WCS, score range: 0 to 15); and stress at work (SAW, score range: 0 to 10) [36]. For each dimension, higher score values denote better QWL. A full-scale score, ranging from 0 to 115, can also be calculated as the sum of the six factor scores. The 24^th^ item of the scale, which explores NSHW’s satisfaction with the overall quality of their working life, is not used in the calculation of factor scores.

### Study population

The study population included survey participants who filled out the WRQoL scale.

### Statistical analyses

Data were weighted and calibrated (calibration on margins using the raking ratio method) to be representative of the whole population of the 12,000 NSHW working in the AP-HP hospitals in terms of sex, age (using 5-year age classes), and professional category (nurses, nurse assistants and laboratory technicians, executives, midwives, and other categories). Descriptive statistics were used to document NSHW’s answers to the questionnaire items and the distribution of QWL scores in the whole study population. Comparisons were then performed between professional categories using chi-square tests for categorical variables and Wald tests for continuous ones. Lastly, weighted linear regression models were used to identify correlates of the WRQoL full-scale score. Variables with a p-value <0.25 in the univariable analyses were considered eligible for the multivariable model. A backward selection procedure was used to build the final multivariable model, which included only statistically significant variables (p<0.05). The Stata version 14.2 for Windows software (StataCorp, College Station, Texas, USA) was used for the analyses.

## Results

### Characteristics of the study population

The study population included 1,387 individuals, and mainly comprised nurses (52.3%). The other professional categories represented were nurse assistant or technicians (38.2%), midwives (4.2%), executives (0.8%), and other categories (4.6%). The latter group included different professions such as reception agents, administrative staff, or pharmacists.

NSHW in the study population were mostly women (77.5%). Mean age (standard deviation, SD) was 39.3 (12.0 years), 54.2% of NSHW were living with a partner, and 50.2% had children (Table 1A). Fourteen percent of NSHW reported facing financial difficulties. Three quarters (75.8%) had a permanent night position and 61.2% worked in a hospital department for adult care. The mean (SD) seniority as a night-shift worker was 9.0 (8.5) years (Table 1B). Socio-demographic, economic, and work-related characteristics differed significantly between professional categories of NSHW (Table 1A to 1E).

**Table 1:** Main characteristics of night-shift healthcare workers according to their professional category (n = 1,387, AP-HP ALADDIN survey, Paris public hospitals).

		Professional category of NSHW					
Characteristics	Whole study population	Nurses	Assistant nurses or technicians	Midwives	Executives	Other categories	*p-value* ^*1*^
	(n = 1,387)	(52.3%)	(38.2%)	(4.2%)	(0.8%)	(4.6%)	
	**Percent [95% CI** ^**♦**^ **] or mean (SD)**						
**a. Socio-demographic and economic characteristics**							
**Female gender**	77.5 [75.1–79.9]	82.4	71.2	96.1	71.2	58.4	***<0*.*001***
**Age—** *in years*	39.3 (12.0)	36.5 (10.7)	43.4 (10.3)	33.1 (8.0)	51.8 (9.5)	40.3 (11.7)	***<0*.*001***
**Matrimonial status**							***<0*.*001***
• single	36.6 [34.0–39.2]	43.2	29.4	29.2	13.1	32.8	
• in cohabitation	20.8 [18.6–23.0]	21.1	18.9	35.3	16.6	20.9	
• in civil partnership or married	33.4 [30.8–35.9]	28.8	39.7	35.6	43.8	29.4	
• widow or widower	9.2 [7.6–10.9]	7.0	12.1	0	26.5	16.9	
**Has children**							***<0*.*001***
• no	49.8 [47.1–52.6]	60.9	33.3	67.8	24.9	49.9	
• yes, and at least one lives at home	42.4 [39.7–45.1]	36.9	52.1	32.2	49.5	32.7	
• yes, but none at home	7.8 [6.1–9.5]	2.2	14.7	0	25.6	17.4	
**Has partial or complete custody of at least one child**	40.5 [37.8–43.1]	36.0	47.8	32.2	47.6	36.3	***<0*.*001***
**Perceived financial status**							***<0*.*001***
• Feels financially comfortable/it’s okay	40.0 [37.3–42.7]	44.9	28.9	84.5	62.0	32.8	
• Has to be careful	46.0 [43.3–48.7]	46.1	50.1	15.5	35.4	40.5	
• Faces financial difficulties	14.0 [11.9–16.0]	9.0	21.0	0	2.6	26.7	
**b. Work-related characteristics**							
**Type of position**							***<0*.*001***
• Permanent night position	75.8 [73.3–78.2]	76.1	84.7	4.9	84.1	61.2	
• Replacement (“pool”)	4.3 [3.2–5.4]	4.4	4.9	0	0	3.7	
• Position with day/night alternation	16.2 [14.1–18.3]	16.7	8.0	95.1	3.8	9	
• New night-shift position during the pandemic	0.8 [0.3–1.3]	0.9	0.9	0	0	0	
• Other	2.9 [1.7–4.1]	2.0	1.5	0	12.1	26.2	
**Hospital department**							***<0*.*001***
• Pediatric	15.1 [13.2–17.1]	16.9	14.7	4.9	1.4	10.5	
• Adults	61.2 [58.5–63.9]	66.5	60.6	46.6	23.9	25.8	
• Several departments*	23.7 [21.3–26.0]	16.6	24.7	48.5	74.7	63.7	
**Hospital unit**							***<0*.*001***
• Surgery	16.4 [14.3–18.5]	16.7	15.4	37.2	9.3	3.7	
• Geriatrics/Rehabilitation	8.8 [7.2–10.5]	6.9	14.0	0	3.3	0	
• Internal medicine/Infectiology/Cardiology/Pneumology	7.3 [6.0–8.6]	9.1	5.8	0	1.2	7.3	
• Neurology/Nephrology/Oncology/Endocrinology	6.5 [5.2–7.8]	8.7	5.1	0	2.4	0	
• Pediatrics	15.7 [13.7–17.7]	17.3	15.8	4.9	1.4	10.5	
• Resuscitation	13.4 [11.7–15.2]	17.9	10.2	0	0	3.6	
• Emergency	7.2 [5.7–8.7]	6.5	7.4	9.4	7.6	11.1	
• Several units*	24.5 [22.1–27]	16.9	26.5	48.5	74.7	63.7	
**Seniority as a night-shift worker** *—in years*	9.0 (8.5)	8.4 (8.1)	9.5 (8.0)	9.7 (6.1)	14.3 (10.6)	7.8 (9.2)	***<0*.*001***
**Daily duration of work**							***<0*.*001***
• 10 hours	62.1 [59.5–64.8]	61.3	72.7	0	80.2	38.1	
• 12 hours	34.0 [31.4–36.6]	36.2	25.1	96.6	8.0	29.9	
• other	3.9 [2.6–5.2]	2.5	2.3	3.4	11.8	32.0	
**Part-time work**	5.2 [4.0–6.4]	5.7	4.3	9.1	7.3	3.2	*0*.*466*
**Travel time to work (home-work one-way commute)—** *in minutes*	42 (36)	42 (24)	48 (36)	48 (54)	36 (24)	48 (24)	***0*.*035***
**c. Health-related characteristics**							
**Perceived health**							***0*.*019***
• Bad or very bad	8.3 [6.7–9.9]	8.5	7.4	4.9	7.7	17.4	
• Fair	40.5 [37.7–43.2]	40.2	41.8	25.1	40.9	47.0	
• Good or excellent	51.3 [48.5–54]	51.3	50.8	70.1	51.4	35.6	
**Practice of any physical activity**	54.2 [51.2–57.1]	52.5	53.4	85.5	39.2	52.8	***<0*.*001***
**Perception of a change in weight since working at night** (309 missing values)	68.2 [65.3–71.1]	68.1	69.8	45.0	68.1	70.6	***0*.*251***
**History of cancer**	3.8 [2.6–5.1]	3.5	3.4	3.4	4.6	12.5	***0*.*026***
**History of psychiatric troubles (depression, bipolar disorders, etc.)**	5.3 [3.9–6.7]	4.1	5.1	7.8	10.9	17.6	***0*.*001***
**History of sexual or moral harassment** **at work**	20.9 [18.5–23.4]	20.6	21.7	12.1	39.6	23.3	*0*.*323*
**History of SARS-CoV-2 infection**							***0*.*014***
• No	58.7 [56.0–61.4]	60.0	60.3	43.3	59.2	44.2	
• Yes	13.6 [11.7–15.5]	14.1	13.3	9.6	14.0	14.5	
• Did not answer	27.7 [25.2–30.2]	25.9	26.4	47.1	26.8	41.3	
**d. Work-related perceptions**							
**Night-shift work is often or always under-estimated by colleagues working during the day**^**2**^	64.7 [62.0–67.4]	67.1	69.6	28.0	73.6	29.9	***<0*.*001***
**Night-shift work is often or always under-estimated by loved ones**^**2**^^,^^**3**^	21.9 [19.6–24.2]	24.3	17.6	36.8	14.0	17.4	***0*.*003***
**Night-shift work is often or always under-estimated by patients** ^**2**^	18.3 [16.2–20.4]	20.2	16.9	12.2	18.2	13.6	*0*.*322*
**Day missions are more important than night missions**^**4**^	24.0 [21.7–26.3]	23.9	25.2	12.7	17.0	27.6	*0*.*271*
**Day workload is higher than night workload** ^**4**^	38.5 [35.8–41.2]	42.7	33.7	18.7	17.7	52.8	***<0*.*001***
**Work rhythm is a source of tension with partner or children** (30.9% in the “not concerned” category)	47.2 [43.1–51.3]	56.8	33.1	79.5	32.2	54.0	***<0*.*001***
**Work rhythm is a source of tension with friends**	20.1 [17.9–22.4]	24.3	11.6	34.4	27.0	28.4	***<0*.*001***
**Feels more irritable since works at night**	43.6 [40.7–46.5]	48.7	33.6	72.8	30.1	40.3	***<0*.*001***
**e. Changes in work organization since the beginning of the COVID-19 pandemic**							
**No change at all **	36.8 [34.1–39.5]	32.5	42.8	25.4	23.2	49.8	***<0*.*001***
**Change of department**	25.8 [23.5–28.2]	27.8	27.3	2.8	14.3	14.0	***<0*.*001***
**Change of ward (part of department)**	29.8 [27.3–32.2]	33.2	31.1	0	13.6	10.8	***<0*.*001***
**Increase of the no. of working hours**	37.2 [34.6–39.9]	39.3	29.0	71.8	65.5	45.5	***<0*.*001***
**Switch to night-shift work**	5.2 [3.9–6.6]	5.0	4.4	4.9	5.6	15.9	***0*.*005***
**Change of activity to manage COVID patients**	19.0 [16.9–21.1]	23.7	15.2	14.4	8.1	3.9	***<0*.*001***
**f. COVID-related items**							
**Satisfied of the information on COVID received from the employer** ^**5**^	31.5 [29.0–34.0]	30.2	32.2	34.1	54.1	34.5	*0*.*440*
**Feels vulnerable to COVID-19 because of professional activity** ^**6**^	77.8 [75.3–80.3]	80.4	73.7	89.4	63.0	72.9	***0*.*015***
**Fears to get the COVID-19 at work**	65.5 [62.7–68.3]	64.2	67.0	73.7	44.3	65.0	*0*.*368*
**Fears to transmit the COVID-19 to close relatives**	90.6 [88.9–92.4]	90.4	90.5	96.5	79.3	91.3	*0*.*477*
**Has received psychological support from close relatives during the previous two weeks**	7.0 [5.4–8.5]	7.6	7.4	0	2.0	4.3	*0*.*286*
**Has received psychological support from a professional during the previous two weeks**	8.4 [6.7–10.1]	8.4	9.6	0	3.3	8.1	*0*.*236*
**Felt valued by the general population as a NSHW during the pandemic**	62.9 [60.1–65.8]	65.0	59.2	76.9	63.0	55.8	*0*.*067*
**Is confident in the health authorities to manage the crisis** ^**6**^	19.6 [17.3–22.0]	18.6	20.4	23.1	43.1	17.7	*0*.*346*
**Faced difficulties in applying protective measures against COVID** ^**6**^	59.7 [56.8–62.6]	59.2	58.9	69.1	44.9	65.9	*0*.*454*
**Considers protective measures inadequate**^**6**^	27.6 [24.9–30.2]	27.6	28.5	23.6	10.9	25.7	*0*.*727*
**Faced difficulties in getting screened for SARS-CoV-2 infection**^**6**^	58.4 [55.5–61.4]	57.8	62.3	56.9	37.3	38.8	***0*.*013***

CI = confidence interval; NSHW = night-shift healthcare workers; SD = standard deviation.

♦ For the purpose of readability of the table, 95% confidence intervals are only presented for the characteristics of the whole study population.

* Concerns healthcare workers assigned to different departments or units.

^1^ Comparison of characteristics between the five professional categories of NSHW (Chi-square tests for categorical variables, Wald test for continuous variables).

^2^ The other possible answers to this item of the questionnaire included “never”, “rarely”, and “from time to time”.

^3^ Loved ones included partner, family, and friends.

^4^ “I totally agree” or “I agree” (*versus* “I totally disagree” or “I disagree”).

^5^ “The information on protective measures against COVID that I received from my employer were sufficient and complete.”

^6^ “I totally agree” or “I agree” (*versus* “I totally disagree”, “I disagree”, or “no interest”).

#### Health-related characteristics

In the whole study population, 51.3% perceived their health as good or excellent, and 54.2% reported physical activity, with highest rates among midwives for these two characteristics (Table 1C). Twenty-one percent (20.9%) of NSHW had faced sexual or moral harassment at work. Nearly fourteen percent of NSHW (13.6%) reported they had contracted COVID-19, but 27.7% did not answer the corresponding item of the questionnaire.

#### Work-related perceptions

Globally, 64.7% of NSHW perceived that night-shift work was often or always under-estimated by their colleagues working during day, and this percentage was highest among executives (Table 1D). Concerning the social consequences of work, 47.2% of NSHW with a partner or children reported work rhythm was a source of tension between one another, 20.1% of NSHW reported work rhythm as a source of tension with friends, and 43.6% felt more irritable since they worked at night. The percentages were highest among midwives for these three characteristics.

#### Changes in work organization since the beginning of the COVID-19 pandemic

Regarding work organization, 36.8% of NSHW reported no change since the beginning of the COVID-19 pandemic, a percentage that was highest in the “other categories” group (Table 1E). Both globally and almost consistently within professional categories, the changes more often reported were increase in the number of working hours (globally 37.2% of NSHW) and change of ward (part of department dedicated to a given specialty) (29.8%). Less than one percent (0.8%) had a new night-shift position since the beginning of COVID-19. Nineteen percent of NSHW changed activity to manage COVID-19 patients, and this percentage was highest among nurses.

#### COVID-related items

NSHW’s responses to the COVID-19 items of the questionnaire showed that most NSHW (77.8%) felt vulnerable to COVID-19 because of their professional activity and 90.6% of them feared to transmit the virus to close relatives (Table 1F). About one third (31.5%) reported that the information their employer gave them on COVID-19 was sufficient and complete, 58.4% faced difficulties in getting screened, 59.7% reported difficulties in applying protective measures against COVID-19, which 27.6% considered inadequate. A total of 19.6% of NSHW felt confident in the health authorities’ ability to manage the crisis. Finally, while 62.9% felt valued by the general population as a NSHW during the pandemic, 7.0% and 8.4% reported having received recent psychological support from close relatives and professionals, respectively (Table 1). No significant difference between professional categories were found concerning NSHW’s responses to the COVID-related items of the questionnaire, except for the percentage of NSHW who felt vulnerable to COVID-19 due to their professional activity (highest among midwives) and that facing difficulties in getting screened for SARS-CoV-2 infection (highest among assistant nurses or technicians).

### Distribution of QWL scores

**Fig 1:**
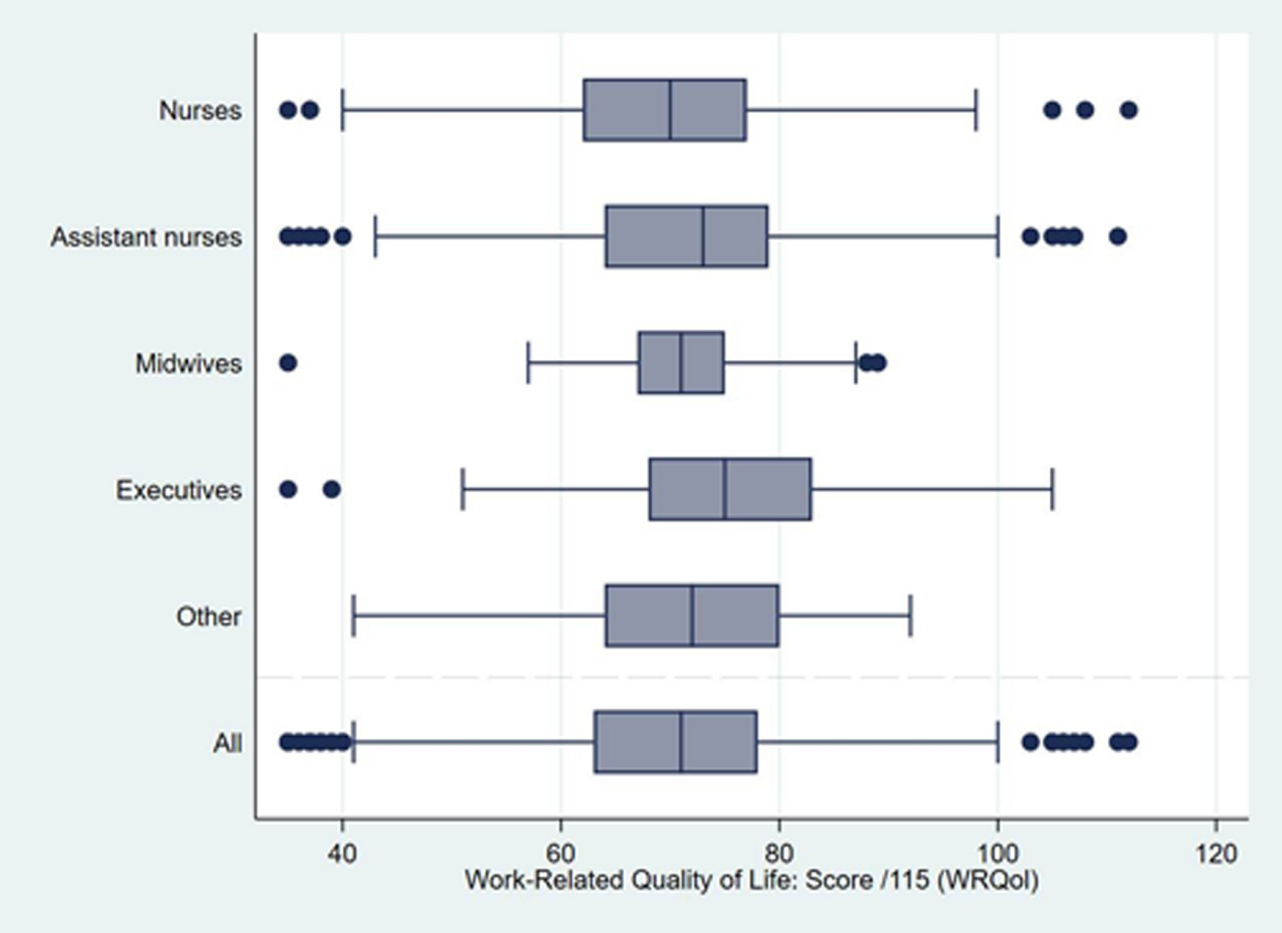
Boxplots of quality of working life scores among night-shift healthcare workers according to their professional category (n = 1,387, AP-HP ALADDIN survey, Paris public hospitals). The boxplots present median values and interquartile ranges (box) for the full-scale WRQoL score (range 0 to 115). Lines (whiskers) include all points within 1.5 interquartile range of the nearest quartile. Higher score values denote better QWL.

Median [interquartile range, IQR] WRQoL full-scale score was 71 [63–78] in the whole study population (Fig 1). Its distribution was significantly different between professional categories, with the highest score observed amongst executives, and the lowest amongst nurses (in mean (SD): 73 (5.8) *versus* 69.6 (10.6), p = 0.001) (Table 2). The distributions of scores for the six dimensions of QWL are also presented in Table 2. Except for general well-being, in addition to job and career satisfaction, significant differences—globally below 1 or 2 points in median, with a maximum of 3 points—were observed between the QWL scores for the different professional categories. Midwives had the lowest QWL scores for home-work interface, working conditions, and stress at work (meaning impaired QWL for these three dimensions) along with the highest QWL score (meaning better QWL) for control at work. Executives presented higher scores in the “Home-work interface” and “Job and career satisfaction” dimensions of QWL, compared with the other professional categories.

**Table 2:** Mean quality of working life scores among night-shift healthcare workers according to their professional category (n = 1,387, AP-HP ALADDIN survey, Paris public hospitals).

Scores calculated from the WRQoL scale^1^ [35] (range)		Professional category of NSHW					
	Whole study population (n = 1,387)	Nurses (52.3%)	Assistant nurses or technicians (38.2%)	Midwives (4.2%)	Executives (0.8%)	Other categories (4.6%)	*p-value* ^*2*^
	mean (SD)						
**GLOBAL WORK-RELATED QUALITY OF LIFE SCORE**							
**Full-scale WRQoL score** (0 to 115)	70.5 (12.0)	69.6 (10.6)	71.7 (14.2)	70.2 (15.7)	73.0 (5.8)	70.9 (20.1)	***0*.*001***
**SCORES ASSOCIATED WITH THE SIX DIMENSIONS OF WORK-RELATED QUALITY OF LIFE**							
**General well-being (GWB)** (0 to 30)	19.3 (4.2)	19.2 (3.8)	19.5 (4.9)	19.3 (5.3)	19.2 (1.9)	19.5 (7.1)	*0*.*844*
**Home-work interface (HWI)** (0 to 15)	9.2 (2.1)	9 (1.9)	9.4 (2.4)	8.4 (2.9)	9.6 (1.0)	9.6 (2.7)	***<0*.*001***
**Job and career satisfaction (JCS)** (0 to 30)	18.8 (3.8)	18.7 (3.2)	18.9 (4.5)	19.4 (6.2)	20.4 (1.8)	18.6 (7.2)	*0*.*060*
**Control at work (CAW)** (0 to 15)	9.3 (2.6)	9.2 (2.3)	9.4 (3.0)	10.3 (3.5)	9.9 (1.1)	8.7 (4.7)	***0*.*025***
**Working conditions (WCS)** (0 to 15)	8.2 (2.6)	8.0 (2.4)	8.5 (2.9)	7.7 (3.1)	8.8 (1.2)	8.7 (3.9)	***0*.*021***
**Stress at work (SAW)** (0 to 10)	5.7 (1.9)	5.6 (1.7)	6.0 (2.1)	5.1 (2.4)	5.2 (0.9)	5.9 (3.0)	***0*.*002***

NSHW = night-shift healthcare workers; IQR = interquartile range; SD = standard deviation; WRQoL = work-related quality of life.

^1^ For each score, higher values denote better quality of working life.

^2^ Comparison of mean scores between the five professional categories of NSHW (Wald test).

### Determinants of QWL

In the multivariable QWL model, satisfaction on the information on COVID received from the employer and feeling valued by the general population as a NSHW during the pandemic were identified as independent correlates of higher full-scale WRQoL score (Table 3), after adjustment for socio-economic characteristics (matrimonial status, professional category, financial difficulties, hospital unit of assignment), perceived health, history of harassment at work, and physical activity.

**Table 3:** Factors associated with quality of working life among night-shift healthcare workers: Linear regression models with full-scale WRQoL score as the outcome (n = 1,387, ALADDIN survey, Paris public hospitals).

	Univariable models			Multivariable model (n = 1,124)		
Characteristics	Coefficient	[95% CI]	*p-value*	Adjusted coefficient	[95% CI]	*p-value*
**SOCIO-DEMOGRAPHIC AND ECONOMIC CHARACTERISTICS**						
**Matrimonial status**						
• single	ref	ref		ref	ref	
• in cohabitation	0.51	[-1.55; 2.56]	0.627	1.03	[-0.68; 2.74]	0.238
• in civil partnership or married	1.17	[-0.54; 2.87]	0.181	0.18	[-1.32; 1.68]	0.814
• widow or widower	3.37	[0.97; 5.76]	0.006	2.45	[0.09; 4.81]	**0.042**
**Perceived financial status**						
• Feels financially comfortable/it’s okay	ref	ref		ref	ref	
• Has to be careful	-3.58	[-5.09; -2.07]	<0.001	-2.07	[-3.49; -0.65]	**0.004**
• Faces financial difficulties	-6.65	[-9.05; -4.26]	<0.001	-4.87	[-7.17; -2.58]	**<0.001**
**WORK-RELATED CHARACTERISTICS**						
**Professional category**						
• Nurses	ref	ref		ref	ref	
• Assistant nurses or technicians	2.08	[0.52; 3.63]	0.009	1.93	[0.50; 3.37]	**0.008**
• Midwives	0.53	[-3.99; 5.05]	0.819	0.13	[-4.06; 4.33]	0.950
• Executives	3.38	[-0.21; 6.98]	0.065	1.68	[-1.18; 4.54]	0.250
• Other categories	1.30	[-3.68; 6.27]	0.609	0.62	[-3.36; 4.60]	0.760
**Type of position**						
• Permanent night position	ref	ref				
• Replacement (“pool”)	-2.70	[-7.01; 1.62]	0.220	-2.33	[-5.28; 0.63]	0.123
• Position with day/night alternation	-0.32	[-2.02; 1.38]	0.709	-1.87	[-4.10; 0.36]	0.100
• New night-shift position during the COVID pandemic	-13.43	[-25.37; -1.50]	0.027	-12.56	[-23.81; -1.31]	**0.029**
• Other	-0.02	[-5.07; 5.04]	0.995	-0.37	[-4.76; 4.02]	0.869
**Hospital unit**						
• Surgery	ref	ref		ref	ref	
• Geriatrics/Rehabilitation	4.96	[1.79; 8.12]	0.002	2.92	[0.22; 5.63]	**0.034**
• Internal medicine/Infectiology/Cardiology/Pneumology	0.82	[-2.27; 3.92]	0.602	1.03	[-1.71; 3.76]	0.461
• Neurology/Nephrology/Oncology/Endocrinology	2.00	[-1.22; 5.21]	0.224	1.80	[-1.07; 4.67]	0.218
• Pediatrics	2.89	[0.20; 5.58]	0.035	1.33	[-0.99; 3.65]	0.262
• Resuscitation	2.24	[0.04; 4.43]	0.046	3.00	[0.89; 5.11]	**0.005**
• Emergency	1.09	[-2.14;4.32]	0.508	1.48	[-1.24; 4.21]	0.286
• Several units	1.87	[-0.21; 3.95]	0.077	1.32	[-0.66; 3.31]	0.191
**HEALTH-RELATED CHARACTERISTICS**						
**Perceived health**						
• Bad or very bad	ref	ref		ref	ref	
• Fair	7.39	[4.20; 10.57]	<0.001	4.98	[2.20; 7.76]	**<0.001**
• Good or excellent	13.56	[10.4; 16.72]	<0.001	8.80	[5.99; 11.61]	**<0.001**
**Practice of any physical activity**	3.54	[2.05; 5.03]	<0.001	1.33	[0.08; 2.57]	**0.037**
**History of sexual or moral harassment at work**	-5.36	[-7.36; -3.35]	<0.001	-3.70	[-5.42; -1.98]	**<0.001**
**WORK-RELATED PERCEPTIONS**						
**Night-shift work is often or always under-estimated by colleagues working during the day** ^**1**^	-3.95	[-5.57; -2.32]	<0.001	-3.54	[-5.01; -2.08]	**<0.001**
**Work rhythm is a source of tension with friends**	-7.11	[-9.08; -5.14]	<0.001	-2.28	[-4.14; -0.42]	**0.016**
**Feels more irritable since works at night**	-5.46	[-6.92; -3.99]	<0.001	-2.96	[-4.34; -1.58]	**<0.001**
**WORK ORGANIZATION: CHANGES SINCE THE BEGINNING OF THE COVID PANDEMIC**						
**COVID-RELATED ITEMS**						
**Satisfied of the information on COVID received from the employer** ^**4**^	7.36	[5.86; 8.85]	<0.001	4.67	[3.26; 6.07]	**<0.001**
**Felt valued by the general population as a NSHW during the pandemic**	3.12	[1.52; 4.71]	<0.001	1.41	[0.13; 2.70]	**0.031**
**Considers protective measures inadequate** ^**5**^	-5.28	[-6.98; -3.57]	<0.001	-2.09	[-3.52; -0.65]	**0.004**
**Faced difficulties in getting screened for SARS-CoV-2 infection** ^**5**^	-4.38	[-5.89; -2.88]	<0.001	-2.95	[-4.25; -1.65]	**<0.001**

CI = confidence interval; WRQoL = work-related quality of life.

♦ This variable was not entered in the multivariable analysis due to a high rate of NSHW in the “not concerned” category.

^1^ The other possible answers to this item of the questionnaire included “never”, “rarely”, and “from time to time”.

^2^ Loved ones included partner, family, and friends.

^3^ “I totally agree” or “I agree” (*versus* “I totally disagree” or “I disagree”).

^4^ “The information on protective measures against COVID that I received from my employer were sufficient and complete.”

^5^ “I totally agree” or “I agree” (*versus* “I totally disagree”, “I disagree”, or “no interest”).

By contrast, a new night-shift position during the pandemic, under-estimation of night-shift work by colleagues working during the day, work rhythm as a source of tension with friends, feeling more irritable since working at night, considering protective measures against the COVID-19 inadequate, and having faced difficulties in getting screened for SARS-CoV-2 infection were all independent correlates of lower full-scale WRQoL score.

## Discussion

This representative survey offers a comprehensive picture of perceived quality of life at work among NSHW in Paris hospitals shortly after the first wave of the COVID-19 pandemic. After adjustment for socio-demographic, professional, and health-related characteristics, both social and professional recognition of night-shift work appeared as key determinants of QWL in this population. By contrast, lack of or insufficient access to screening, information, and protective measures significantly impaired QWL.

These findings highlight the impact on QWL of the difficulties faced by hospital teams to organize the chain of information and to provide safety equipment to all caregivers during the first wave of COVID-19, despite their preparedness and training for emergency situations. Indeed, this unexpected global health crisis caused by a previously unknown virus has deeply challenged healthcare workers’ adaptability [37], and has stressed the need to update safety guidelines to protect and prevent infection in hospital workers [38]. Another study in the COVID-19 context underlined that coping strategies could influence healthcare workers’ well-being and QWL [39]. Findings from ALADDIN also highlight the importance of recognizing the contribution of all healthcare workers [37]. Previous work showed that emphasizing the value of healthcare workers’ role was essential to motivate them and to increase their willingness to work during public health emergency situations [40]. Professional recognition also includes feeling supported by peers. In the ALADDIN survey, 64.7% of NSHW reported that night-shift work is often or always under-estimated by colleagues working during the day, and this perceived stigma had a significant detrimental effect on QWL. These findings highlight the need to develop interventions to improve communication, sharing of experiences, and support between day-shift and night-shift hospital healthcare workers. Such interventions can reinforce the sense of community among healthcare workers, and have the potential to improve NSHW’s experience in the workplace. In addition, findings confirm that healthcare workers’ perception of their public image can influence their QWL [41].

Results from the univariable analyses confirm the detrimental effect on QWL of self-perceived vulnerability to COVID-19 and fear of transmitting the infection to close relatives. Previous research has also shown a negative psychological impact of these two factors among healthcare workers in France [42]. Interestingly, in ALADDIN, these factors were not identified as independent correlates of QWL in the final multivariable model, maybe because of their correlation with other COVID-related variables such as difficulties to get screened and perceived inadequate and insufficient protective measures. In the same way, changes in work organization since the beginning of the pandemic did not remain in the model after multivariable adjustment.

There is a lack of published studies on QWL conducted among healthcare workers, especially in France. We identified only one recent survey, also based on the WRQoL scale [43]. QWL level observed in ALADDIN was lower than that found in this recent survey, conducted among 2,040 French anesthesiologists (median [IQR] WRQoL full-scale score: 71 [63–78] *versus* 77 [66–85]) [43]. This difference is likely to be related to the study period, as the latter survey was performed before the beginning of the COVID-19 pandemic (January to June 2019). It may also be related to the diversity of professional categories participating in ALADDIN, presenting different levels of QWL.

Compared with the WRQoL scale’s norms, the median QWL score in ALADDIN corresponds to a relatively low level of QWL. However, these norms refer to the UK National Health Services [36], and may not be adapted to the French context because of differences between countries in the organization and functioning of healthcare services. Cultural specificities may also play a role, as shown in other research areas such as perception of happiness [44]. These specificities may be linked to differences in people’s work-related representations and expectancies. Environmental factors such as the socio-political context in different countries may make international comparisons even more difficult.

Findings from ALADDIN showed statistically significant differences in QWL between professional categories. These differences were however of modest magnitude and did not exceed 3 points in QWL scores. Further research is needed to determine if such a magnitude exceeds the minimum important difference for the WRQoL scale. Executives showed both the best overall QWL and higher scores in the “Home-work interface” and “Job and career satisfaction” dimensions of QWL, compared with that of other professional categories. Along with older age, correlated with less domestic responsibilities related to child care, a longer experience of night-shift work may explain the greater ability of executives to find the right balance between their professional and personal lives. By contrast, executives (together with midwives) presented a low score of QWL in the “Stress at work” dimension, revealing higher levels of stress than other professional categories. Interestingly, midwives reported the lowest QWL related to working conditions. Further research should thus be performed to identify midwives’ specific needs and expectations to both improve their QWL and prevent psychosocial risks [45]. Of note, the number of years in night-shift work (variable “seniority as a night-shift worker (in years)”) was not significantly associated with overall QWL, despite its heterogeneity in our study sample. We hypothesize that seniority may influence one’s night-shift work experience in different ways. For instance, workers with more night-shift work experience may better cope with stress than those with less experience. By contrast, the latter may have been less exposed to changes in the circadian rhythm, resulting in better perceived health.

The ALADDIN survey has several strengths. First, its representativeness regarding sex, age, and professional categories allows presenting a snapshot of QWL among all NSHW working in Paris public hospitals. Second, the choice of the study period, which directly followed the first wave of COVID-19 in France (March to June 2020), is adequate to assess NSHW’s perceptions during the pandemic. Indeed, once their work overload started to decline after the peak of the crisis, NSHW were more prone to both share their feelings and experiences, and to assess the repercussions of the pandemic on their QWL. Lastly, the ALADDIN survey explores a large panel of potential correlates of QWL, using a standard scale (WRQoL).

However, the survey is limited by its cross-sectional design. Further research is therefore needed to assess longitudinal changes in QWL among NSHW throughout the pandemic, and in the long term. Another limitation of our study is the lack of comparative data among day-shift hospital workers. Such data would have helped distinguish between the effects of shift-work by itself on QWL and those related to coping with the pandemic. Future surveys should include both populations of hospital workers. Of note, external factors such as the time of day the questionnaire was completed may have influenced NSHW’s answers (notably due to fatigue). This type of bias, inherent to self-reported data, is difficult to take into account in the analyses. Indeed, a potential “time of the day” effect depends on many unmeasured factors, including NSHW’s number of hours worked before completing the questionnaire, their workload, and inter-individual variations in the internal clock (some individuals feel awake late at night, whilst others are sleepy).

In France, there is a growing interest for healthcare professionals’ quality of life at work, with a national strategy for the improvement of QWL (“Caring the caregivers”), aiming notably at improving work environment and work conditions, informing managers about QWL-related issues and psychosocial risks, and supporting them in the adoption of better work methods [46]. In line with this strategy, a national observatory was created in 2018 to monitor QWL among healthcare and medico-social workers. The COVID-19 pandemic has further stressed the need to document QWL in healthcare services, and to identify its determinants during and after such sanitary crises [47]. Findings from the AP-HP ALADDIN survey contribute to increase the body of knowledge about these key issues, which are central to set up efficient strategies to reinforce healthcare systems. Such strategies should include interventions aiming to improve recognition, reduce stigma related to night-shift work, and to improve information and communication between the different groups of healthcare workers.

To conclude, in this representative survey, insufficient access to screening, information, and protective measures impaired QWL of NSHW after the first wave of COVID-19 in Paris public hospitals. Social and professional recognition of night-shift work appear as key determinants of QWL in this population. Further research is needed to monitor longitudinal changes in QWL of NSHW during and after the different waves of the COVID-19 pandemic.

## References

[1] Rydz E, Hall AL, Peters CE. Prevalence and Recent Trends in Exposure to Night Shiftwork in Canada. *Ann Work Expo Health* 2020; 64: 270–281. doi: 10.1093/annweh/wxaa001 32020159

[2] Cordina-Duverger E, Houot M, Tvardik N, et al. Prévalence du travail de nuit en France: caractérisation à partir d’une matrice emplois-expositions. [in French] http://invs.santepublique france.fr/beh/2019/8-9/2019_8–9_3.htm. *Bull Epidémiol Hebd* 2019; (8–9): 168–174.

[3] Trades Union Congress (TUC). Number of people working night shifts up by more than 150,000 in 5 years. https://www.tuc.org.uk/news/number-people-working-night-shifts-more-150000-5-years

[4] Foster RG. Sleep, circadian rhythms and health. *Interface Focus* 2020; 10: 20190098. doi: 10.1098/rsfs.2019.0098 32382406 PMC7202392

[5] Mohd Azmi NAS, Juliana N, Mohd Fahmi Teng NI, et al. Consequences of Circadian Disruption in Shift Workers on Chrononutrition and their Psychosocial Well-Being. *Int J Environ Res Public Health*; 17. Epub ahead of print 19 March 2020. doi: 10.3390/ijerph17062043 32204445 PMC7142532

[6] Walker WH, Walton JC, DeVries AC, et al. Circadian rhythm disruption and mental health. *Transl Psychiatry* 2020; 10: 28. doi: 10.1038/s41398-020-0694-0 32066704 PMC7026420

[7] Chellappa SL, Vujovic N, Williams JS, et al. Impact of Circadian Disruption on Cardiovascular Function and Disease. *Trends Endocrinol Metab* 2019; 30: 767–779. doi: 10.1016/j.tem.2019.07.008 31427142 PMC6779516

[8] Haus EL, Smolensky MH. Shift work and cancer risk: potential mechanistic roles of circadian disruption, light at night, and sleep deprivation. *Sleep Med Rev* 2013; 17: 273–284. doi: 10.1016/j.smrv.2012.08.003 23137527

[9] Fagundo-Rivera J, Gómez-Salgado J, García-Iglesias JJ, et al. Relationship between Night Shifts and Risk of Breast Cancer among Nurses: A Systematic Review. *Medicina (Kaunas)*; 56. Epub ahead of print 10 December 2020. doi: 10.3390/medicina56120680 33321692 PMC7764664

[10] Esmaily A, Jambarsang S, Mohammadian F, et al. Effect of shift work on working memory, attention and response time in nurses. *Int J Occup Saf Ergon* 2020; 1–19. 10.1080/10803548.2020.1863656 33308103

[11] D’Oliveira TC, Anagnostopoulos A. The Association Between Shift Work And Affective Disorders: A Systematic Review. *Chronobiol Int* 2020; 1–19. doi: 10.1080/07420528.2020.1838533 33222534

[12] Szkiela M, Kusideł E, Makowiec-Dąbrowska T, et al. How the Intensity of Night Shift Work Affects Breast Cancer Risk. *Int J Environ Res Public Health*; 18. Epub ahead of print 26 April 2021. doi: 10.3390/ijerph18094570 33925799 PMC8123502

[13] Khosravipour M, Khanlari P, Khazaie S, et al. A systematic review and meta-analysis of the association between shift work and metabolic syndrome: The roles of sleep, gender, and type of shift work. *Sleep Med Rev* 2021; 57: 101427. doi: 10.1016/j.smrv.2021.101427 33556868

[14] Gehlert S, Clanton M. Shift Work and Breast Cancer. *Int J Environ Res Public Health*; 17. Epub ahead of print December 2020. doi: 10.3390/ijerph17249544 33419321 PMC7767214

[15] Zhang Q, Chair SY, Lo SHS, et al. Association between shift work and obesity among nurses: A systematic review and meta-analysis. *Int J Nurs Stud* 2020; 112: 103757. doi: 10.1016/j.ijnurstu.2020.103757 32921429

[16] Fink AM. Measuring the effects of night-shift work on cardiac autonomic modulation: an appraisal of heart rate variability metrics. *Int J Occup Med Environ Health* 2020; 33: 409–425. doi: 10.13075/ijomeh.1896.01560 32427129

[17] Alfonsi V, Scarpelli S, Gorgoni M, et al. Sleep-Related Problems in Night Shift Nurses: Towards an Individualized Interventional Practice. *Front Hum Neurosci* 2021; 15: 644570. doi: 10.3389/fnhum.2021.644570 33796014 PMC8007770

[18] Boivin DB, Boudreau P. Impacts of shift work on sleep and circadian rhythms. *Pathol Biol (Paris)* 2014; 62: 292–301. 25246026 10.1016/j.patbio.2014.08.001

[19] Torquati L, Mielke GI, Brown WJ, et al. Shift work and the risk of cardiovascular disease. A systematic review and meta-analysis including dose-response relationship. *Scand J Work Environ Health* 2018; 44: 229–238. doi: 10.5271/sjweh.3700 29247501

[20] Turchi V, Verzuri A, Nante N, et al. Night work and quality of life. A study on the health of nurses. *Ann Ist Super Sanita* 2019; 55: 161–169. doi: 10.4415/ANN_19_02_08 31264639

[21] Nena E, Katsaouni M, Steiropoulos P, et al. Effect of Shift Work on Sleep, Health, and Quality of Life of Health-care Workers. *Indian J Occup Environ Med* 2018; 22: 29–34. doi: 10.4103/ijoem.IJOEM_4_18 29743782 PMC5932908

[22] Kim W, Kim TH, Lee T-H, et al. The impact of shift and night work on health related quality of life of working women: findings from the Korea Health Panel. *Health Qual Life Outcomes* 2016; 14: 162. doi: 10.1186/s12955-016-0564-x 27894317 PMC5126815

[23] Ruzevicius J. *Quality of Life and of Working Life*: *Conceptions and Research*. 2014.

[24] Morin E. Sens du travail, santé mentale et engagement organisationnel. Rapport R-543 [Internet]. Montréal, Québec: Institut de recherche Robert-Sauvé en santé et en sécurité to travail du Québec; 2008. http://www.irsst.qc.ca/media/documents/pubirsst/r-543.pdf

[25] Vilas Boas AA. Quality of life and quality of working life. *Edited by Ana Alice Vilas Boas* *(*2017*)*. *Published*: *August 23rd 2017*. *ISBN*: 978-953-51-3446-6. *Ed*. *IntechOpen*.

[26] Pratt MG, Ashforth BE. *Fostering meaningfulness in working and at work*, *In* Cameron K. S., Dutton J. E. *&* Quinn R. E *(Eds)*. *Positive Organizational Scholarship*: *Foundations of a New Discipline*. San Francisco: Berret-Koehler, 309–327. 2003.

[27] Haute Autorité de santé (HAS). Revue de littérature. Qualité de vie au travail et qualité des soins [in French]. Janvier 2016. doi: 10.1016/j.soin.2016.03.004

[28] Rizza S, Coppeta L, Grelli S, et al. High body mass index and night shift work are associated with COVID-19 in health care workers. *Journal of endocrinological investigation*. Epub ahead of print May 2021. doi: 10.1007/s40618-020-01397-0 32852704 PMC7450678

[29] Buselli R, Corsi M, Baldanzi S, et al. Professional Quality of Life and Mental Health Outcomes among Health Care Workers Exposed to Sars-Cov-2 (Covid-19). *Int J Environ Res Public Health* 2020; 17: E6180. doi: 10.3390/ijerph17176180 32858810 PMC7504107

[30] Duracinsky M, Cousin L, Marcellin F, et al. Management of the COVID-19 health crisis: perceptions and experience of night-shift healthcare workers during the first wave of the pandemic in Paris public hospitals (the AP-HP ALADDIN survey). IAS COVID-19 conference: Prevention—2 February 2021—e-poster n°255.

[31] Duracinsky M, Cousin L, Coscas S, et al. Vécu et gestion de la crise sanitaire liée à la Covid-19: le point de vue du personnel hospitalier de nuit de l’Assistance publique–Hôpitaux de Paris durant la première vague épidémique (enquête AP-HP Aladdin, 15 juin-15 septembre 2020). ht tp://beh.santepubli quefrance.fr/ beh/2021/Cov_6/2021_Cov_6_1.html. *Bull Epidémiol Hebd*; (Cov_6):2–9.

[32] Van Brakel WH. Measuring health-related stigma—a literature review. *Psychol Health Med* 2006; 11: 307–334. doi: 10.1080/13548500600595160 17130068

[33] Berger BE, Ferrans CE, Lashley FR. Measuring stigma in people with HIV: psychometric assessment of the HIV stigma scale. *Res Nurs Health* 2001; 24: 518–529. doi: 10.1002/nur.10011 11746080

[34] Golay P, Moga M, Devas C, et al. Measuring the paradox of self-stigma: psychometric properties of a brief scale. *Ann Gen Psychiatry* 2021; 20: 5. doi: 10.1186/s12991-021-00325-7 33468180 PMC7814463

[35] Van Laar D, Edwards JA, Easton S. The Work-Related Quality of Life scale for healthcare workers. *J Adv Nurs* 2007; 60: 325–333. doi: 10.1111/j.1365-2648.2007.04409.x 17908128

[36] Easton S, Van Laar D. Work-Related Quality of Life (WRQoL) Scale—A Measure of Quality of Working Life- First edition, University of Portsmouth, 2013.

[37] Mehta S, Machado F, Kwizera A, et al. COVID-19: a heavy toll on health-care workers. *Lancet Respir Med* 2021; 9: 226–228. doi: 10.1016/S2213-2600(21)00068-0 33556317 PMC7906726

[38] Ferioli M, Cisternino C, Leo V, et al. Protecting healthcare workers from SARS-CoV-2 infection: practical indications. *Eur Respir Rev* 2020; 29: 200068. doi: 10.1183/16000617.0068-2020 32248146 PMC7134482

[39] McFadden P, Ross J, Moriarty J, et al. The Role of Coping in the Wellbeing and Work-Related Quality of Life of UK Health and Social Care Workers during COVID-19. *International journal of environmental research and public health*; 18. Epub ahead of print 19 January 2021. doi: 10.3390/ijerph18020815 33477880 PMC7832874

[40] Hope K, Durrheim D, Barnett D, et al. Willingness of frontline health care workers to work during a public health emergency. *Australian Journal of Emergency Management* 2010; 25: 39–47.

[41] Roshangar F, Soheil A, Moghbeli G, et al. Iranian nurses’ perception of the public image of nursing and its association with their quality of working life. *Nursing open*. Epub ahead of print 5 May 2021. doi: 10.1002/nop2.892 33951343 PMC8510743

[42] Chene G, Nohuz E, Cerruto E, et al. Psychological impact on healthcare workers in obstetrics and gynecology in France in 18 French University Hospitals during the first Covid-19 lockdown: a prospective observational study. *J Psychosom Obstet Gynaecol* 2021; 1–8. 10.1080/0167482X.2021.2014812 34915826

[43] Gafsou B, Becq M, Michelet D, et al. Determinants of Work-Related Quality of Life in French Anesthesiologists. *Anesthesia and analgesia*. Epub ahead of print 2 April 2021. doi: 10.1213/ANE.0000000000005397 33543868

[44] Senik C. The French Unhappiness Puzzle: the Cultural Dimension of Happiness. halshs-00628837v6. 2014; 106: 379–401.

[45] Cramer E, Hunter B. Relationships between working conditions and emotional wellbeing in midwives. *Women Birth* 2019; 32: 521–532. doi: 10.1016/j.wombi.2018.11.010 30578019

[46] French Ministry of Health. Stratégie nationale d’amélioration de la qualité de vie au travail—Prendre soin de ceux qui nous soignent [in French]- 5 Décember 2016. https://solidarites-sante.gouv.fr/IMG/pdf/strategie_qvt_2016.pdf

[47] French National Observatory for Quality of working life among healthcare and medico-social professionals. Qualité de vie au travail et COVID 19—Contribution de l’Observatoire national de la qualité de vie au travail des professionnels de santé et du médico-social—Repères pour les pratiques -15 décembre 2020 [in French].

