# Correction: Social and professional recognition are key determinants of quality of life at work among night-shift healthcare workers in Paris public hospitals (AP-HP ALADDIN COVID-19 survey)

**DOI:** 10.1371/journal.pone.0308720

**Published:** 2024-08-08

**Authors:** Martin Duracinsky, Fabienne Marcellin, Lorraine Cousin, Vincent Di Beo, Véronique Mahé, Olivia Rousset-Torrente, Patrizia Carrieri, Olivier Chassany

There are errors in the author affiliations. The correct affiliations are as follows:

Martin Duracinsky^1,2,3^, Fabienne Marcellin^4^, Lorraine Cousin^2,3,4^, Vincent Di Beo^4^, Véronique Mahé^5^, Olivia Rousset-Torrente^2,3^, Patrizia Carrieri^4^, Olivier Chassany^2,3^

**1** Département de médecine interne et d’immunologie clinique, hôpital Bicêtre, AP-HP, Kremlin-Bicêtre, France, **2** Université de Paris, Patient-Reported Outcomes Unit (PROQOL), UMR 1123, Inserm, Paris, France, **3** Unité de recherche clinique en économie de la santé (URC-ECO) AP-HP, hôpital Hôtel-Dieu, Paris, France, **4** Aix Marseille Univ, Inserm, IRD, SESSTIM, Sciences Economiques & Sociales de la Santé et Traitement de l’Information Médicale, ISSPAM, Marseille, France **5** Service central de santé au travail, hôpitaux Lariboisière-Fernand Widal, AP-HP Nord, Paris, France
