# Supplementary material for: Social and professional recognition are key determinants of quality of life at work among night-shift healthcare workers in Paris public hospitals (AP-HP ALADDIN COVID-19 survey)
Source: PLoS One. 2022 Apr 7;17(4):e0265724. doi: 10.1371/journal.pone.0265724 (PMC9045406; doi:10.1371/journal.pone.0265724)
Supplement: Attachment — Submitted filename: Response to reviewers 20janv2022.docx [file pone.0265724.s001.docx]

Fabienne MARCELLIN

INSERM UMR1252- SESSTIM

Faculté de Médecine

3e étage - Aile bleue

27 boulevard Jean Moulin

13385 Marseille cedex 5 FRANCE

e-mail: [fabienne.marcellin@inserm.fr](mailto:fabienne.marcellin@inserm.fr)

Marseille, 20 January 2022

**To Prof. Florian Fischer, Academic Editor, PLoS One**

Dear Editor,

Thank you very much for giving us the opportunity to submit a revised version of our manuscript entitled “*Social and professional recognition are key determinants of quality of life at work among night-shift healthcare workers in Paris public hospitals (AP-HP ALADDIN COVID-19 survey)”* to PLoS One.

Below, please find detailed responses to the reviewers’ comments.

As requested, we have attached a Microsoft Word version of the revised manuscript that highlights changes made to the original version, and a final unmarked version of the revised manuscript.

We hope that this revised version will meet the criteria for publication in PLoS One, and we remain available to make any changes which could further improve our manuscript.

Best regards,

Fabienne MARCELLIN, corresponding author

**Reviewers' comments:**

**Reviewer's Responses to Questions**

**Comments to the Author**

**1. Is the manuscript technically sound, and do the data support the conclusions?**

The manuscript must describe a technically sound piece of scientific research with data that supports the conclusions. Experiments must have been conducted rigorously, with appropriate controls, replication, and sample sizes. The conclusions must be drawn appropriately based on the data presented.

Reviewer #1: Partly

Reviewer #2: No

We have complemented the discussion of our results, as suggested by the reviewers (please see answers to reviewers’ comments in point 5).

**2. Has the statistical analysis been performed appropriately and rigorously?**

Reviewer #1: No

Reviewer #2: Yes

We have performed the complementary statistical analyses asked by the reviewers (please see answers to reviewers’ comments in point 5).

**3. Have the authors made all data underlying the findings in their manuscript fully available?**

The PLOS Data policy requires authors to make all data underlying the findings described in their manuscript fully available without restriction, with rare exception (please refer to the Data Availability Statement in the manuscript PDF file). The data should be provided as part of the manuscript or its supporting information, or deposited to a public repository. For example, in addition to summary statistics, the data points behind means, medians and variance measures should be available. If there are restrictions on publicly sharing data—e.g. participant privacy or use of data from a third party—those must be specified.

Reviewer #1: No

Reviewer #2: Yes

A data sharing statement has been added in the revised manuscript, as follows:

***“DATA SHARING STATEMENT***

***Data is available upon request to the scientific committee of the ALADDIN survey.”***

**4. Is the manuscript presented in an intelligible fashion and written in standard English?**

PLOS ONE does not copyedit accepted manuscripts, so the language in submitted articles must be clear, correct, and unambiguous. Any typographical or grammatical errors should be corrected at revision, so please note any specific errors here.

Reviewer #1: Yes

Reviewer #2: No

The manuscript has been re-read and copyedited by two native US English speakers.

**5. Review Comments to the Author**

Please use the space provided to explain your answers to the questions above. You may also include additional comments for the author, including concerns about dual publication, research ethics, or publication ethics. (Please upload your review as an attachment if it exceeds 20,000 characters)

**Reviewer #1: Overview:**

The authors evaluated a questionnaire of determinants of quality of life at work among night-shift healthcare workers in Paris public hospitals. The central idea of evaluating the quality of life of night workers during the first wave of COVID is very interesting. Both for the impact on the biological rhythms of night work and for the challenges of working on the front lines during this period of pandemic. The idea is therefore very commendable.

We thank the reviewer for this positive feedback.

However, the great challenge of this work should be the demonstration with subjective and perhaps objective data, which are the factors that negatively impact the quality of life inherent to night work (eg: factor "x" = night work) plus the factors that coping with the pandemic itself, regardless of the work shift, is also negatively affected (eg factor "y" = COVID pandemic). This distinction is not clearly described and analyzed in this manuscript.

An example: they could also have analyzed the day shift workers in the same period and compared the data. We would have a more accurate view of the “pandemic x work shift” effect.

We agree with the reviewer concerning the difficulties to distinguish between the effects of shift-work by itself and those related to coping with the pandemic. Our model was adjusted for factors related to each of these two domains (characteristics and organization of work, perceptions, and experience since the beginning of the pandemic), leading to an estimation of each effect independently of the others.

As noted by the reviewer, it would have been interesting to compare the level and correlates of quality of life at work between day-shift workers and night-shift workers. This should be explored in future surveys.

We have noted this as a limitation of our study in the Discussion section of the revised manuscript, as follows:

***“Another limitation of our study is the lack of comparative data among day-shift hospital workers. Such data would have helped distinguish between the effects of shift-work by itself on QWL and those related to coping with the pandemic. Future surveys should include both populations of hospital workers.”***

**Minor concerns**

The worker's experience in the night shift should be considered and analyzed separately. Workers with less than three weeks on the night scale will not show the same effects perceived as "negative" by workers with one year on the night scale. Likewise, workers with “a lot” of time on the night shift schedule could already be more physically and psychologically tolerant to typical circadian changes and, in many cases, also feeling less of these effects.

This is an interesting point. We have already described the variable “Seniority as a night-shift worker,” and tested it as a potential correlate of quality of life at work.

As shown by the univariable analyses, it was not significantly associated with the full-scale WRQoL score (coefficient [95% confidence interval: 0.01 [-0.09; 0.11], p=0.811), even if hospital workers’ years of experience with night-shift work varied greatly (mean (standard deviation) of seniority as a night-shift worker: 9 (8.5) years).

We have discussed this point in the revised manuscript, as follows:

***“Of note, the number of years in night-shift work (variable “seniority as a night-shift worker (in years)”) was not significantly associated with overall QWL, despite its heterogeneity in our study sample. We hypothesize that seniority may influence one’s night-shift work experience in different ways. For instance, workers with more night-shift work experience may better cope with stress than those with less experience. By contrast, the latter may have been less exposed to changes in the circadian rhythm, resulting in better perceived health.”***

**Major concerns**

The time of day when the questionnaires were answered also influence the results. From a chronobiological point of view, a night shift worker who answered an online questionnaire at 5 pm, after a period of rest, will likely have a different score if the same worker answers the same questionnaire at 5 am, after your work’s turn. This should be described and considered in this manuscript.

In the analyses, it is difficult to take into account the time of day when the questionnaire was filled in. Indeed, the study sample includes individuals with different work schedules (some having permanent night positions, others working alternatively between day and night shift). In addition, we have not collected information on the number of hours worked and the global workload of survey participants prior to completing the questionnaire. A worker completing the questionnaire at 2 a.m. may feel exhausted from a non-stop 4-hour rush, while another may be in good shape, if activity is calm in the department. Inter-individual variations in the internal clock further complexify the interpretation of a possible “time of the day” effect (some people feeling comfortable and awake even late at night, and others feeling sleepy at the same period).

We have added a point in the Discussion section of the revised manuscript, as follows:

***“Of note, external factors such as the time of day the questionnaire was completed may have influenced NSHW’s answers (notably due to fatigue). This type of bias, inherent to self-reported data, is difficult to take into account in the analyses. Indeed, a potential “time of the day” effect depends on many unmeasured factors, including NSHW’s number of hours worked before completing the questionnaire, their workload, and inter-individual variations in the internal clock (some individuals feel awake late at night, whilst others are sleepy).”***

All results were described in tables only. Graphic resources with joint presentation as well as correlation data described separately would be essential.

We have added a graphical representation of QWL scores using boxplots, as shown below. This enabled us to alleviate data presented in Table 2 (please see revised manuscript).

**Figure 1 -** **Boxplots of quality of working life scores among night-shift healthcare workers according to their professional category (n=1,387, AP-HP ALADDIN survey, Paris public hospitals)**

***The boxplots present median values and interquartile ranges (box) for the full-scale WRQoL score (range 0 to 115). Lines (whiskers) include all points within 1.5 interquartile range of the nearest quartile. Higher score values denote better QWL.***

All data in tables is overly descriptive and difficult to visualize. It can be confusing for the reader to try to compare data presented at the beginning with data at the end of the table.

We agree that the presentation of descriptive statistics in Table 1 may be difficult to read due to the high number of lines. Consequently, we have split the data into six different sub-tables, each representing one group of variables (from “Sociodemographic and economic characteristics” to “COVID-related items”). This will facilitate the interpretation of the tables, while keeping information on each variable analyzed available to readers.

In addition, in Table 3, we have deleted information about variables which did not remain in the final multivariable model.

We have also changed double-spacing to 1.5-line spacing in all tables.

History of psychiatric troubles (depression, bipolar disorders) seems to be a factor that directly contributes to the subjective answers (questionnaires) related to quality of life. In this case, it would be interesting for the authors to also present this data separately, considering the presence or absence of this factor in the other results.

The sample size of NSHW with a history of psychiatric troubles was too small in our survey (n=60) to perform a stratified analysis. Indeed, the lack of statistical power and unbalanced distribution of the variable “history of psychiatric troubles” (4.3% “yes” versus 95.7% “no” in the dataset of the multivariable model) do not enable neither the correct identification of QWL correlates nor the comparison of odds ratios between the two groups.

**Discussion:**

The title of the manuscript focuses on the results of workers' recognition during the pandemic as keys to improving quality of life. However, this data (recognition) was mentioned only 4 times in the entire manuscript, and it was not properly discussed.

We have added a description of variables related to social and professional recognition in the Methods section of the revised manuscript, as follows (Data collection paragraph):

***“NSHW’s perceptions regarding their social and professional recognition were assessed using items related to under-estimation of night-shift work by colleagues, loved ones, and patients; perceptions of the importance of night missions and of workload during night; feeling valued by the general population as a NSHW during the pandemic. Most of these items were derived from different stigma scales (Brakel WHV 2006, Berger BE et al. 2001, Golay P et al. 2021)”.***

We have also enriched the discussion of workers’ recognition during the pandemic, as follows:

***“Professional recognition also includes feeling supported by peers. In the ALADDIN survey, 64.7% of NSHW reported that night-shift work is often or always under-estimated by colleagues working during the day, and this perceived stigma had a significant detrimental effect on QWL. These findings highlight the need to develop interventions to improve communication, sharing of experiences, and support between day-shift and night-shift hospital healthcare workers. Such interventions can reinforce the sense of community among healthcare workers, and have the potential to improve NSHW’s daily experience in the workplace.”***

The feeling of being vulnerable to COVID infection, as well as the fear of transmitting the disease to family members, can be crucial factors in these workers' negative quality of life scores. Still, the sudden change in the organization of work seems to be another impacting factor on quality of life, especially for nurses. These data should be discussed with other results already presented in the literature, when one wants to compare the effects of the pandemic on these professionals, regardless of the work shift.

We chose to focus our discussion on variables which were significantly associated with quality of working life in the multivariable model. Fear of getting infected or to transmit the disease to family members, and changes in work organization have not been identified as independent correlates of quality of working life in the final model.

Nevertheless, in the revised Discussion section, we have added information on these variables, as suggested:

***“Results from the univariable analyses confirm the detrimental effect on QWL of self-perceived vulnerability to COVID-19 and fear of transmitting the infection to close relatives. Previous research has also shown a negative psychological impact of these two factors among healthcare workers in France (Chene G et al 2021). Interestingly, in ALADDIN, these factors were not identified as independent correlates of QWL in the final multivariable model, maybe because of their correlation with other COVID-related variables such as difficulties to get screened and perceived inadequate and insufficient protective measures. In the same way, changes in work organization since the beginning of the pandemic did not remain in the QWL model after multivariable adjustment.”***

It would be essential, at least in the discussion, to describe what the literature presented as "quality of life", with these same analysis tools, of workers before the pandemic (control). The work of Gafsou B, et. al, 2021 could have been more discussed. Again, the data were only presented descriptively. What was expected is an intense discussion of each variable.

To our knowledge, the only previous study which used the WRQoL scale in the French context was of the one by Gafsou et al, which was conducted before the COVID pandemic and can thus be used as a reference.

We have underlined the lack of published data, as follows (Discussion section):

***“There is a lack of published studies on QWL conducted among healthcare workers, especially in France. We identified only one recent survey, which was also based on the WRQoL scale (Gafsou et al).”***

The authors commented,"Cultural specificities may also play a role, as shown in other research areas such as perception of happiness" and that the WRQoL scale's norms is a British instrument (UK) and may not be It is possible to compare with the French context, due to cultural differences between countries. However, to assess the QWL the WRQol scale was used. In other hand, was used this same instrument for the population of French workers. Wouldn't that be contradictory and difficult to trust the data presented?

The use of the WRQoL scale is justified here, as it is a standard and validated psychometric tool that enables comparisons between past studies conducted in specific subgroups of French healthcare workers (such as anesthesiologists in the work by Gafsou et al.) as well as with future studies assessing QWL (in healthcare contexts or in other professional contexts).

However, comparing the level of QWL between individuals from different countries remains difficult because of differences in people’s work-related representations and expectancies (what we called “cultural specificities”), and differences in environmental factors such as the social and political context. Of note, UK and France share common characteristics, as both are Northern, high-resource countries of the European geographic region.

We have added the following sentences in the discussion of results:

***“These specificities may be linked to differences in people’s work-related representations and expectancies. Environmental factors such as the socio-political context in different countries may make international comparisons even more difficult.”***

Finally, in order to affirm the effects of the pandemic on shift workers, in addition to the cross-sectional portrait, the authors are enhanced to explore the longitudinal analysis of the data, so that we can comprehensively understand the impacts of this period on this specific population of workers.

Unfortunately, data collected are cross-sectional. Even if a “longitudinal” dimension has been explored in certain items of the questionnaire (such as variables related to changes in work organization since the beginning of the pandemic), future studies are needed to better understand the impact of the COVID period among NSHW, especially in the long term.

We have modified one sentence in the limitations section of the revised manuscript as follows:

*“However, the survey is limited by its cross-sectional design. Further research is therefore needed to assess longitudinal changes in QWL among NSHW throughout the pandemic****, and in the long term.****”*

**Reviewer #2:**

This paper aimed to document the determinants of quality of working life (QWL) among night-shift healthcare workers (NSHW) in Paris public hospitals after the first-wave of the COVID-19 pandemic. However, there are few issues that were unclear to me and need further clarification, as below:

1. The use of “several department”, “several unit” are unclear to me. Suggestion for the authors is to explain these variable under footnote.

The categories “several departments” and “several units” include NSHW assigned to different departments or units (for instance, people working both in adult and pediatric departments).

We have added the following footnote in Table 1b:

***“* Concerns healthcare workers assigned to different departments or units.”***

2. AP-HP is not spelled out in abstract.

This has been corrected.

3. The significance of this study is not clearly provided.

We have better highlighted the significance of the study at the beginning of the abstract, as follows:

*“****Documenting the perceptions and experiences of frontline healthcare workers during a sanitary crisis is key to reinforce healthcare systems. We identify*** *the determinants of quality of working life (QWL) among night-shift healthcare workers (NSHW) in public hospitals in Paris shortly after the first-wave of the COVID-19 pandemic.”*

4. No information is given on the first wave of pandemic? Why the assessment was done after first wave? What is the significance of having this study after first wave?

In France, the first wave of the COVID-19 pandemic lasted from March to May 2020. The ALADDIN survey was performed shortly after from 15 June to 15 September 2020), which enabled us to reach NSHW after the initial urgent period, once they were less in the heat of the action, more available to answer the survey, and to have a little hindsight to report their perceptions.

We have modified the following sentence of the Methods section:

*“One of the main objectives was to document NSHW’s QWL (i.e. perceived quality of life at work) and its correlates* ***shortly*** *after the first wave of the COVID-19 pandemic* ***(March to May 2020), once healthcare workers were more available to participate in the survey.****”*

5. “The ALADDIN cross-sectional survey (15 June 2020 to 15 September 2020) 76 was conducted among NSHW in the 39 hospitals of the Assistance Publique - Hôpitaux de Paris (AP-HP).” How the hospitals are selected? And any inclusion/ exclusion criterias for the selection?

The survey targeted NSHW in all public hospitals of the Parisian area. It was conducted among all 39 hospitals of the Assistance Publique - Hôpitaux de Paris (AP-HP), with no specific inclusion/exclusion criteria at the level of hospitals.

We have specified this in the Methods section of the revised manuscript, as follows:

*“The ALADDIN cross-sectional survey (15 June 2020 to 15 September 2020) was conducted among NSHW in* ***public hospitals in Paris. It included all 39 hospitals of the Assistance Publique - Hôpitaux de Paris (AP-HP)****.”*

6. What is the response rate? This may lead to information bias when the response rate is too low.

The web survey was available to all NSHW of the AP-HP hospitals. The response rate was approximately 11.5% (1387 /12,000), which was close to our initial objective (1200/12,000). Independently of the response rate, it has to be noted that data weighting and calibration enabled us to work on a representative dataset in terms of age, sex, and professional category.

7. Few of the variables, i.e., “physical activity”, “Change in weight since works at night”, “Travel time to work” are unclear to me. What are the operational definitions for these variables. Table 3: 0.7 hours in the travel time means?

Information on physical activity was collected using the following item:

“Do you practice physical activity?”

Similarly, healthcare workers were asked if they perceived their weight to not have changed, increased or decreased since the beginning of night-shift work.

“Travel time to work” relates to the duration of the home-work one-way commute.

We have modified the label of these variables in the tables, as follows:

*- “Physical activity”* has been changed in ***“Practice of any*** *physical activity****”.***

**- *“****Change in weight since works at night”* has been changed to ***“Perception of a*** *change in weight since working at night”.*

*- “Travel time to work” has been changed to “Travel time to work (****home-work one-way commute)****”.*

0.7 hours of commute means that the NSHW spends nearly three quarters of an hour for a one-way commute. We have converted travel times to minutes in the table.

8. Page 29, Line 238: “social and professional recognition ” is unclear to me. Please explain.

We have added a description of variables related to social and professional recognition in the Methods section of the revised manuscript, as follows (Data collection paragraph):

***“NSHW’s perceptions regarding their social and professional recognition were assessed using items related to under-estimation of night-shift work by colleagues, loved ones, and patients; perceptions of the importance of night missions and of workload during the night; and feeling valued by the general population as a NSHW during the pandemic.”***

We have also enriched the discussion of workers’ recognition during the pandemic, as follows:

***“Professional recognition also includes feeling supported by peers. In the ALADDIN survey, 64.7% of NSHW reported that night-shift work is often or always under-estimated by colleagues working during the day, and this perceived stigma had a significant detrimental effect on QWL. These findings highlight the need to develop interventions to improve communication, sharing of experiences, and support between day-shift and night-shift hospital healthcare workers. Such interventions can reinforce the sense of community among healthcare workers, and have the potential to improve NSHW’s daily experience in the workplace.”***

9. Table 1 is poorly presented.

We have made a short description of each group of variables presented in Table 1. A more detailed description would lead to redundancies between text and tables.

Of note, as recommended by Reviewer 1, we have modified Table 1 to improve readability: it has been split into six different sub-tables, each representing a group of variables. This allows a correspondence with paragraphs in the Results section.

10. Page 30, Line 283: “The lack of reference values for the WRQoL scale in France also limits the discussion of results.”. What is the importance of having reference values in this study?

We refer here to the fact that “WRQoL” norms were developed using data collected in UK National Health Services, and not in France. As this point was already raised earlier in the Discussion, we have removed the sentence page 3 line 283.

11. Page 30, Line 263: “These differences were however of modest magnitude and did not exceed 3 points in QWL scores.” Is 3 points as a cutoff value to determine the difference between groups? Please provide references.

To our knowledge, the minimum important difference in WRQoL scores is not documented among healthcare workers. We have added the following sentence in the Discussion:

***“Further research is needed to determine if such a magnitude exceeds the minimum important difference for the WRQoL scale.”***

12. Page 31, Line 290: “Findings from the AP-HP ALADDIN survey contribute to increase the body of knowledge about these key issues, which are central to set up efficient strategies to reinforce healthcare systems.” What are the examples of strategies that could be recommended by the authors based on the findings of this study.

We have added examples of strategies as follows:

***“Such strategies should include interventions aiming to improve recognition, reduce stigma related to night-shift work, and to improve information and communication between the different groups of healthcare workers.”***

13. The findings of Table 2 is poorly discussed.

Findings in Table 2 (distribution of QWL scores) are difficult to discuss due to a lack of data in the literature. We have raised this point as follows:

***“There is a lack of published studies on QWL conducted among healthcare workers, especially in France. We identified only one recent survey, which was also based on the WRQoL scale (40).*** *QWL level observed in ALADDIN was lower than that found in this recent survey, conducted among 2,040 French anesthesiologists (median [IQR] WRQoL full-scale score: 71 [63-78] versus 77 [66–85]) (40). This difference is likely to be related to the study period, as the latter survey was performed before the beginning of the COVID-19 pandemic (January to June 2019). It may also be related to the diversity of professional categories participating in ALADDIN, presenting different levels of QWL.”*

Other results presented in Table 2 (differences in scores associated with each dimension of QWL) are discussed as follows:

*“Executives showed both the best overall QWL and higher scores in the “Home-work interface” and “Job and career satisfaction” dimensions of QWL, compared with that of other professional categories. Along with older age, correlated with less domestic responsibilities related to childcare, a longer experience of night-shift work may explain the greater ability of executives to find the right balance between their professional and personal lives. By contrast, executives (together with midwives) presented a low QWL score in the “Stress at work” dimension, revealing higher levels of stress than other professional categories. Interestingly, midwives reported the lowest QWL related to working conditions. Further research should thus be performed to identify midwives’ specific needs and expectations to both improve their QWL and prevent psychosocial risks (42).”*
